# Structured light imaging mesoscopy: application to skin changes in scleroderma

**DOI:** 10.1117/1.BIOS.2.2.025002

**Published:** 2025-06-18

**Authors:** Aarohi Mahesh Mehendale, Mahsa Parsanasab, Kavon Karrobi, Hung Vo, Andreea M. Bujor, Vasan Venugopalan, Darren Roblyer

**Affiliations:** a Boston University, Department of Biomedical Engineering, Boston, Massachusetts, United States; b University of California, Irvine, Department of Chemical and Biomolecular Engineering, Irvine, California, United States; c University of California, Irvine, Beckman Laser Institute and Medical Center, Irvine, California, United States; d Boston University Chobanian & Avedisian School of Medicine, Division of Rheumatology, Boston, Massachusetts, United States; e Boston University, Arthritis and Autoimmune Diseases Center, Boston, Massachusetts, United States; f Boston University, Department of Electrical and Computer Engineering, Boston, Massachusetts, United States

**Keywords:** spatial frequency domain imaging, Monte Carlo, scleroderma, fibrosis, diffuse optics

## Abstract

**Significance:**

Scleroderma is a chronic, autoimmune disorder, characterized by the fibrosis of skin and internal organs. The extent of skin fibrosis is a good predictor of disease severity and mortality. Structured light imaging mesoscopy (SLIM) is a noncontact and label-free imaging technique that requires minimal postprocessing and no inverse model. SLIM may provide improved disease diagnosis and monitoring of scleroderma.

**Aim:**

We aim to evaluate SLIM as a new optical method to assess skin involvement in scleroderma.

**Approach:**

SLIM measurements were collected from 25 scleroderma patients and 18 healthy controls across a range of spatial frequencies and wavelengths to find the optimal imaging parameters across skin tones. Monte Carlo simulations were conducted on a multilayer skin model that mimicked known biological changes in scleroderma.

**Results:**

Clinical data and simulations show that optimal spatial frequency ($f_{x}$) and spectral (λ) imaging parameters for scleroderma monitoring lie in the range of $f_{x}=0.15$ to $0.2\;{\mathrm{mm}}^{-1}$ and λ=811 to 851 nm, irrespective of subject skin tone. SLIM measurements taken with these parameters provided discrimination of scleroderma from healthy skin with an area under the curve of ≥0.87. We hypothesize that these changes correspond to changes in dermal collagen.

**Conclusions:**

SLIM can be used as a quantitative and objective method to assess skin involvement in scleroderma. The optimal imaging parameters target the relevant depths of the skin, making it sensitive to biological changes in scleroderma.

Statement of Discovery We introduce the application of a new noncontact optical method, structured light imaging mesoscopy (SLIM) for assessing skin changes in scleroderma. This method requires minimal postprocessing and can be easily clinically translated. SLIM may help clinicians track disease progression and make treatment decisions; it shows potential for validation as an outcome measure for clinical trials.

## 1 Introduction

In part 1 of this two-part publication, structured light imaging mesoscopy (SLIM) was introduced as a new optical technique (part 1 is published in the *Journal of Biomedical Optics*).^1^ This noncontact optical method is relatively simple, requiring just a single spatial illumination pattern, no inverse model, and can probe intermediate depths in multilayer media such as skin. Here, we show that SLIM can be applied for the characterization of an autoimmune disease, scleroderma, that affects the scattering properties of the skin,^2^ particularly in the dermis.^3^

Scleroderma, or systemic sclerosis (SSc), is a multisystem, autoimmune chronic disorder, characterized by fibrosis of the skin and internal organs.^3^ SSc is a rare disease, with a pooled global prevalence of 17.6 individuals per 100,000.^4^ It predominantly affects patients of middle-age, with the mean age at diagnosis being 40 to 50 years old. Approximately 80% of patients are women.^5^ SSc is typically diagnosed with initial symptoms such as fatigue, puffy hands, an exaggerated response to cold (Raynaud’s phenomenon), and joint pain followed by the tightening of the skin. As the disease progresses, other symptoms may include heartburn, pulmonary fibrosis, pulmonary hypertension, and gastrointestinal complications.^3^ Although SSc is a rare disease, it has a considerable impact on those it affects. Internal organ complications, particularly affecting the lungs, are responsible for the mortality associated with this disease. SSc patients have the highest mortality of all autoimmune diseases, surpassing lupus^6^ and cancers such as breast cancer and melanoma,^7^^,^^8^ with a 5-year survival rate of 75% to 80% and a 10-year survival rate of 62% to 65%.^9^ Skin involvement in SSc is clinically manifested by induration, itchiness, and pain, which in turn can lead to loss of mobility and inability to fully open the mouth or extend the fingers, leading to dramatic loss of quality of life. There are no uniformly effective therapies for SSc.

An objective and quantitative metric to assess the disease progression would aid the development of treatment plans and monitoring patient response to therapy. The extent and progression of skin involvement (stiffening and thickening of the skin) in SSc is central to disease classification into subtypes and is a predictor for future organ complications and survival. An increase in skin thickening has been shown to be associated with new or worsening internal organ involvement and increased mortality.^10^^–^^12^

The current clinical gold standard to evaluate the extent of skin involvement in SSc is the modified Rodnan skin score (mRSS). The mRSS is a semi-quantitative, subjective integer score between 0 and 3. It is assigned to 17 different body regions based on clinical palpation of skin. A score of 0 corresponds to no palpable skin induration/thickening, and a score of 3 corresponds to “severe” skin thickening, where there is an inability to make skin folds when the skin is pinched.^13^ mRSS has multiple documented drawbacks, including high interobserver variability, susceptibility to sampling bias, and low sensitivity to longitudinal changes.^14^^,^^15^ A four-point integer scale cannot fully capture the range of skin involvement as skin palpations cannot reliably distinguish between skin thickness/ stiffness and skin tethering, or between the edematous phase and the atrophic phase of the disease.^13^ These limitations hamper accurate quantification of the disease progression and compound the uncertainty of dealing with a rare and challenging disease. In addition, the low reliability and high interobserver variability preclude the effectiveness of mRSS as an outcome measure for clinical trials.

SSc progression usually begins with early vascular injury, which affects small vessels, leading to inflammation and tissue swelling/edema. This is followed by fibrosis and stiffening of the skin, initially noted in the lower dermis, where fibroblasts become activated and deposit large amounts of extracellular matrix. As the disease progresses, there is loss of subcutaneous fat, flattening of the epidermis with loss of rete ridges, and atrophy of dermal appendages. Densely packed collagen across the entire thickness of the dermis characterizes the later stages of the disease.^3^ Changes in collagen architecture in the skin occur across both the papillary and reticular dermis.^16^ Skin changes frequently happen in three sequential phases—first, the edematous phase (characterized by puffy fingers due to edema but with no skin thickening), then the fibrotic or indurative phase (characterized by tight, thickened skin), and finally the atrophic phase (characterized by thinning of skin that is bound down to subcutaneous tissues and immobile).^13^

There is a need for a reliable, quantitative method of tracking skin involvement in SSc. Our group previously proposed using spatial frequency domain imaging (SFDI) as a method of assessing skin involvement in SSc based on a small pilot study.^2^ We measured 10 SSc patients and 8 healthy controls and found that there was a significant decrease in the $\mu_{s}^{\prime }$ values of patients compared with the $\mu_{s}^{\prime }$ values of healthy controls. It was hypothesized in this study that this decrease in $\mu_{s}^{\prime }$ values was due to changes in collagen fiber density and orientation, reduction or displacement of lipids, or loss of tissue differentiation between the epidermal and dermal layers. We also tested the inter- and intraobserver reliability of SFDI and found that it was considerably more reliable when compared with mRSS. These results suggested that SFDI-derived metrics such as $\mu_{s}^{\prime }$ were sensitive to skin changes due to SSc and may provide a more reliable alternative to mRSS.

In part 1, we introduced the concept of SLIM, which extracts diffuse reflectance ($R_{d}$) maps at specific spatial frequencies across a wide field of view to detect subsurface scattering changes in a computational skin model.^1^ The $R_{d}$ map contains information about the tissue microarchitecture within the skin, which we hypothesize is changing during SSc disease progression. The depth sensitivity of SLIM is tunable by adjusting imaging parameters, such as the illumination wavelength and spatial frequency. We hypothesize that an optimal selection of these imaging parameters can provide maximal sensitivity to skin changes during SSc disease progression. As mentioned in Part 1, SLIM does not involve the use of multiple frequencies to resolve an inverse problem and eliminates difficulties associated with spatial assignment of optical properties due to the partial volume effect, which affords it several key advantages compared with SFDI.

In this work, we investigate the efficacy of SLIM as an alternate method to assess skin involvement in SSc. In addition, we select optimized SLIM imaging parameters to target relevant biological changes occurring within the skin as the disease progresses. This is done first by analyzing SLIM data from a clinical study and then with Monte Carlo simulations that mimic clinical SLIM measurements using a multilayer skin model with optical properties perturbed in specific sublayers to mimic sclerodermatous skin.

## 2 Methods

### 2.1 Subject Eligibility and Enrollment

The clinical study was conducted in compliance with an approved institutional review board protocol, applicable regulatory requirements, and BMC/BU Medical Campus Human Research Protection policies and procedures (Protocol Numbers: 38234 and 4698). Subjects were informed about the study and provided informed consent prior to the experiment at the time of regularly scheduled clinic visits. The study was conducted at the Outpatient Rheumatology Clinic at the Boston University Medical Center and at Boston University.

### 2.2 SLIM Technique and Imaging Protocol

**Fig. 1:**
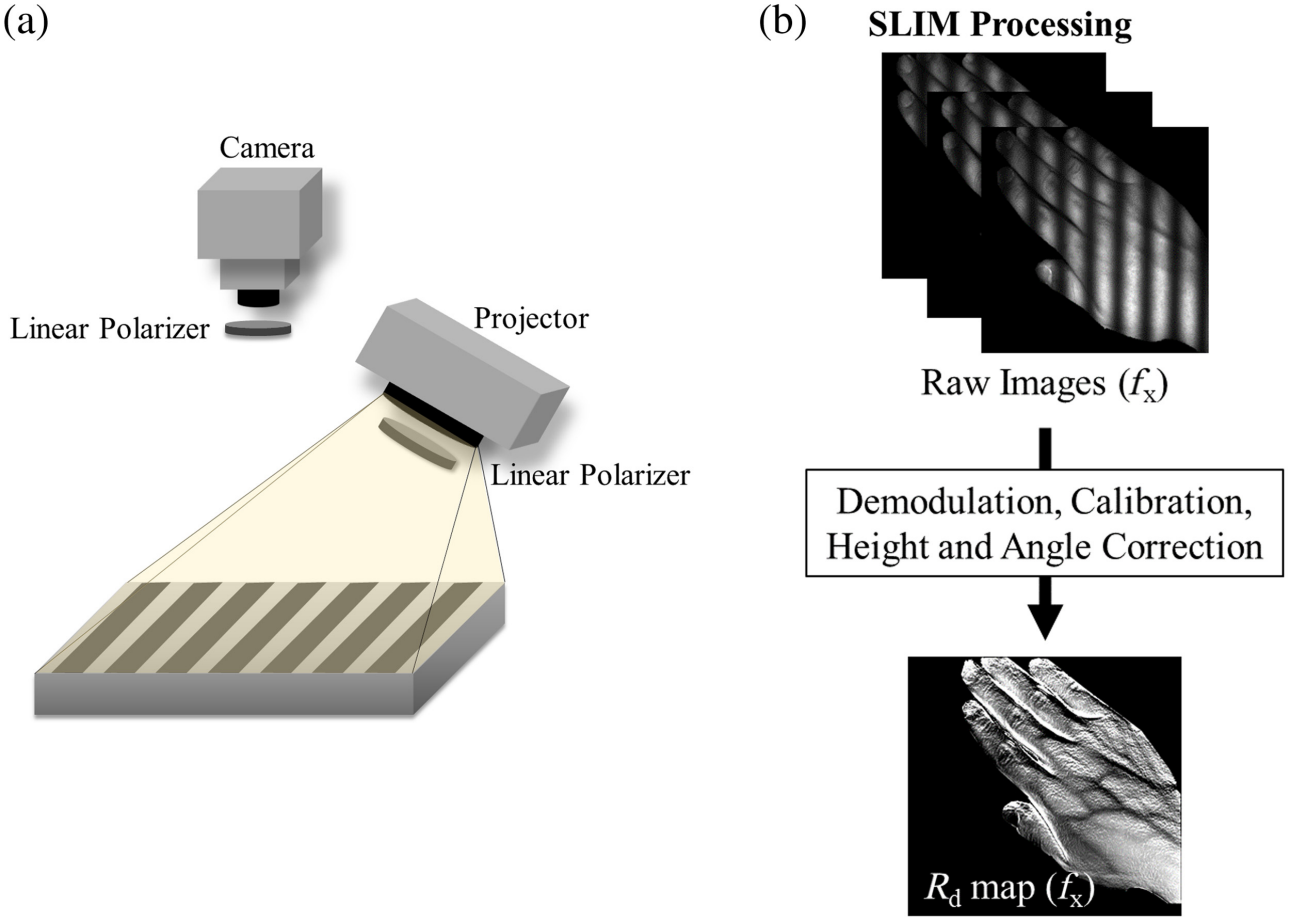
Structured light imaging mesoscopy (SLIM). (a) Schematic diagram of an SLIM system. “Projector” in this diagram refers to the illumination source (a set of visible-near infrared LEDs), combined with a digital micromirror device that projects sinusoidal patterns. Two crossed linear polarizers are placed in the illumination and detection path to avoid specular reflection from the tissue. (b) SLIM data acquisition and processing. AC images are acquired in three phases, these images are demodulated, calibrated, and corrected for height and angle, and a calibrated reflectance map is generated ($R_{d}$). Importantly, unlike SFDI, no inverse model is utilized for SLIM processing, which allows for substantial simplification of processing and hardware.

SLIM provides wavelength and spatial frequency–dependent maps of tissue reflectance over a wide field of view on a pixel-by-pixel basis. A spatial sinusoidal pattern with spatial frequency content is projected on a sample using a digital micromirror device (DMD), and the re-emitted light is collected by a camera [Fig. 1(a)]. The sinusoidal pattern is projected at three phases (0, 120, and 240 deg), and images are collected at each of these phases. These images are demodulated using the following demodulation method (Eq. 1), where $I_{1}$, $I_{2}$, and $I_{3}$ refer to the images in the three phases.

$$I=\;\frac{\sqrt{2}}{3}\sqrt{(I_{1}-I_{2}{)}^{2}+\;(I_{2}-I_{3}{)}^{2}+\;(I_{3}-I_{1}{)}^{2}.}$$ (1)

The demodulated image (I) at each spatial frequency is calibrated against an optical phantom with known and stable optical properties to correct the instrument response. The resulting images at each spatial frequency $f_{x}$ and wavelength λ are maps of calibrated diffuse reflectance ($R_{d}$) [Fig. 1(b)].

In this study, the Reflect RS system from Modulim Inc. (Irvine, California, United States) was used to project the sinusoidal pattern and acquire the three phase-shifted images. SLIM-specific postprocessing was done on the collected images to generate $R_{d}$ maps of the measurement sites. The Reflect RS system uses light-emitting diodes (LEDs) with wavelengths from the visible to the NIR for illumination and has a charge-coupled device (CCD) camera to collect remitted light. The field of view of the images is 15×20  cm. To account for height and angle variabilities within the sample, we performed height and angle correction using a previously developed algorithm.^17^^,^^18^

Subjects were measured at three different body locations, the fingers, dorsal hand, and dorsal forearm, on both their left and right sides. Hand and finger measurements were done concurrently as they both fit within the field of view of the device. Subjects were asked to sit on a chair and place their hand or forearm under the instrument, such that their entire limb was centered in the field of view of the device. Measurements were taken at four wavelengths (691, 731, 811, and 851 nm) and eight spatial frequencies (DC, 0.05, 0.1, 0.15, 0.2, 0.3, 0.4, $0.5\;{\mathrm{mm}}^{-1}$). The four longest imaging wavelengths of the Reflect RS were used based on our preliminary SFDI results.^2^ Profilometry measurements for height and angle correction were performed at 691 nm and $0.05\;{\mathrm{mm}}^{-1}$, based on our previous work.^2^ Each body location measurement (hand/finger together or forearm) takes approximately a minute, and the measurements of all six body locations per subject take ∼5  min.

### 2.3 mRSS Assessment

mRSS assessments were performed by an expert rheumatologist (A.B.) for each subject. mRSS was determined at 17 body locations (right and left fingers, hands, forearms, upper arms, thighs, legs and feet, face, chest, and abdomen). Each body location was assigned an integer score between 0 and 3. The scores corresponded to 0 (normal), 1 (mildly thickened), 2 (moderately thickened), and 3 (severely thickened), as previously published.^13^

### 2.4 Data Analysis

Mean $R_{d}$ values from selected regions of interest (ROIs) were used as representative values for each measurement site. Rectangular ROIs were selected to cover the maximum area of the measurement site. For the forearm, an ROI of dimensions 800×300  pixels (∼11.5×4.3  cm), aligned along the dorsal forearm was used. For the hand, a square ROI of dimensions 300×300  pixels (∼4.3×4.3  cm) on the dorsal side, between the wrist and the knuckles, was used. For the fingers, five ROIs, each of the dimensions of 100×25  pixels (1.44×0.36  cm), were selected between the metacarpophalangeal joint and the proximal interphalangeal joint on the dorsal side of each finger and averaged to provide one representative value. ROIs are shown in Fig. S1 in the Supplementary Material. ROIs were selected in MATLAB (R2021b, The Mathworks, Inc., Natick, Massachusetts, United States).

Separability between SSc patient and healthy control groups was quantified by calculating the area under curve (AUC) under the receiver operating characteristic (ROC) curve of a binary classifier based on a threshold. The $R_{d}$ value from each measurement site from each subject was labeled as either “patient” or “control,” and the sensitivity and specificity were calculated at each different threshold to build an ROC curve. The inbuilt MATLAB function *perfcurve* was used to build this curve and calculate the area under the curve.

Spearman’s correlation coefficient was used to quantify the correlation of total $R_{d}$ values with total mRSS values. The rank-based, nonparametric Spearman’s correlation coefficient was used as mRSS values are noncontinuous. For each subject, a total $R_{d}$ value was calculated by adding the average values across the six measurement sites for that subject. The total mRSS value for each subject was the sum of the corresponding scores of those six measurement sites. AUC values of the separation between SSc patients and healthy control groups and Spearman’s correlation coefficients of the correlation between total $R_{d}$ values and total mRSS values were compared across all wavelength and spatial frequency ($\lambda -f_{x}$) combinations to identify the optimal imaging parameters. All data were analyzed using MATLAB.

### 2.5 Monte Carlo Simulations

Monte Carlo simulations of photon transport were performed on a four-layer skin model. Optical properties were assigned to the epidermis, papillary dermis, reticular dermis, and subcutaneous tissue, based on our previous work.^1^ Two multilayer models were constructed, one “baseline,” which represented the optical properties of “healthy” skin, and another perturbed model, which represented sclerodermatous skin, with optical properties reflecting changes that we hypothesize occur in the skin during disease progression. Specifically, in the perturbed model, the scattering coefficient ($\mu_{s}^{\prime }$) of the papillary and the reticular dermis was decreased by 20%. Given our previous work,^2^ we know that there is a decrease in measured $\mu_{s}^{\prime }$ of the patients compared with healthy controls. The magnitude of the $\mu_{s}^{\prime }$ change seen in our previous work roughly matches the 20% perturbation in this model. In addition, it is known that there are changes in collagen across the full thickness of the dermis.^16^ These changes include changes in orientation, density, and type of collagen, with a preponderance of embryonic collagen,^19^^,^^20^ which has been shown to have a lower scattering coefficient than mature collagen.^21^ Properties of the multilayer skin model are shown in Table 1. Optical properties of the model before and after perturbation are shown in Tables S1 and S2 in the Supplementary Material.

**Table 1:** Properties of multilayer skin model used for Monte Carlo simulations.

Layer	Thickness (μM)	BVF (%)	[Hb] (μM)	[HbO] (μM)	[HbR] (μM)	$H_{2}O$ (%)	Lipid (%)	g	n
Epidermis	50	0	0	0	0	17	10	0.80	1.37
Papillary dermis	200	2	46.51	37.21	9.302	70	5	0.80	1.40
Reticular dermis	1100	2	46.51	37.21	9.302	70	5	0.80	1.40
Subcutaneous tissue	6150	7	162.8	130.2	32.56	70	23	0.75	1.40

Conventional Monte Carlo simulations were performed on both the baseline and perturbed model for four wavelengths and eight spatial frequencies, corresponding to those used in the clinical study. Three epidermal melanin concentrations were considered (2%, 5%, and 10%) using perturbation Monte Carlo^22^^,^^23^ to study the effect of skin color on the optimal ($\lambda \text{-}f_{x}$) combination by changing the optical properties for the epidermis in both the baseline and perturbed models (Table S3 in the Supplementary Material). The open-source MCCL software package (v8.0) was used to perform the Monte Carlo simulations.

## 3 Results

### 3.1 Subject Enrollment

Data were collected from 25 SSc patients and 18 healthy controls. A summary of subject information is shown in Table 2. The local mRSS for the SSc patients ranged from 0 to 3. Subjects self-reported their race as Black, White, or Other, and their ethnicity as Hispanic/Latino and not Hispanic/Latino. The subjects in this data set included the 10 SSc patients and 8 healthy controls measured in our previous study.^2^

**Table 2:** Clinical summary of enrolled subjects.

	Patients	Controls
**Total**	N=25	N=18
**Sex**		
Male	N=6	N=9
Female	N=19	N=9
**Race**		
Black	N=6	N=5
White	N=17	N=9
Other	N=2	N=4
**Ethnicity**		
Hispanic/Latino	N=3	N=5
Non-Hispanic/Latino	N=22	N=13
**Mean age**	49 ± 17 (y.o.)	37 ± 16 (y.o)

### 3.2 Clinical Results

**Fig. 2:**
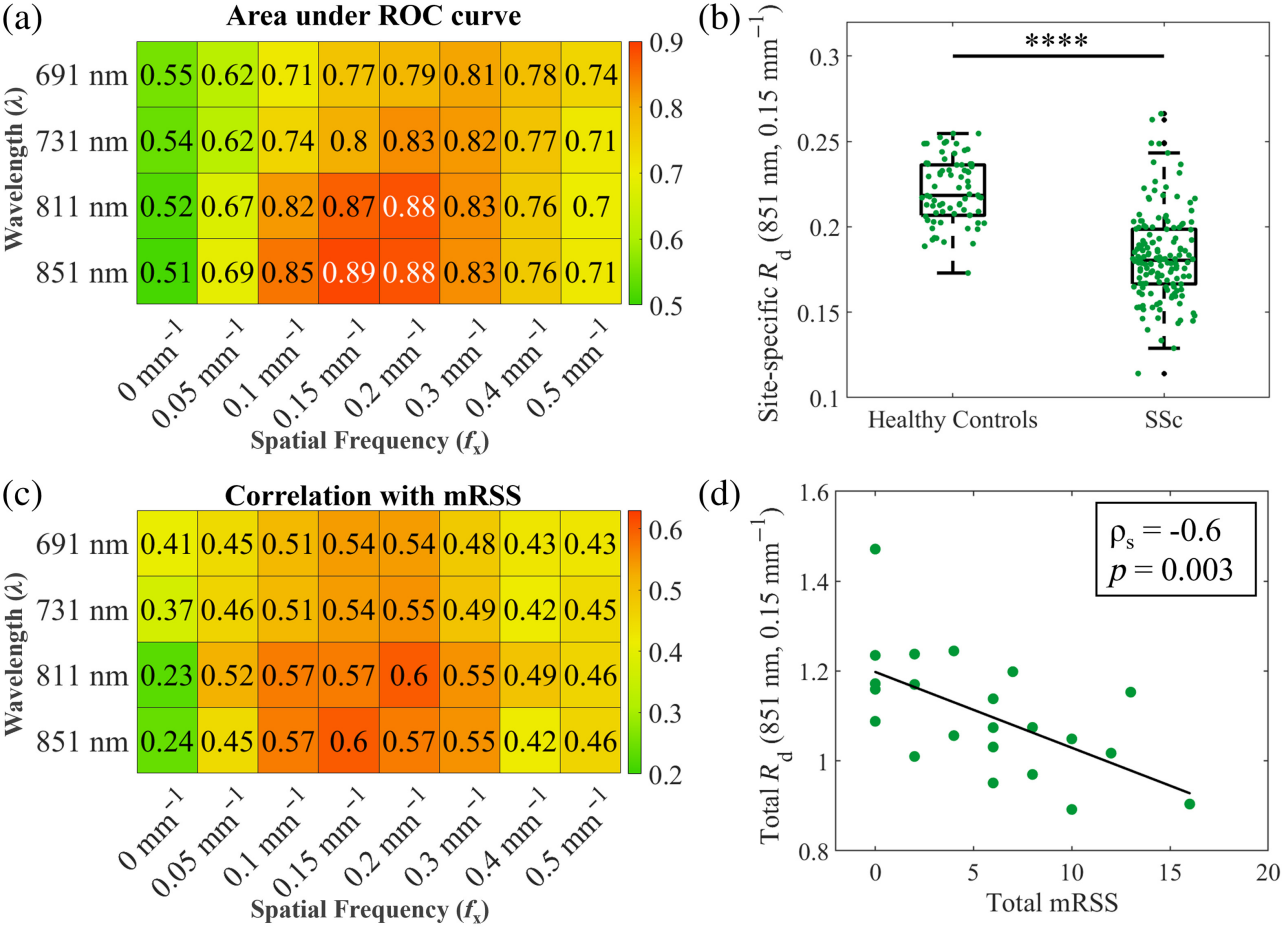
(a) Heatmap of area under ROC curves of threshold classification between site-specific $R_{d}$ values of healthy controls and SSc patients. (b) Example boxplot of site-specific $R_{d}$ values of healthy controls and SSc patients, shown here for a wavelength of 851 nm and spatial frequency of $0.15\;{\mathrm{mm}}^{-1}$. Each point on the boxplot represents the $R_{d}$ value of a different measurement site. (****p<0.0001, Wilcoxon rank sum test). (c) Heatmap of Spearman’s correlation coefficients between total mRSS and total $R_{d}$ across six measured sites. Correlations are calculated between total mRSS and total $R_{d}$. (d) Example correlation plot between total mRSS and total $R_{d}$ at an example wavelength of 851 nm and example spatial frequency of $0.15\;{\mathrm{mm}}^{-1}$. Each point represents one subject where the sum of average $R_{d}$ values (total $R_{d}$) across all measured body sites is plotted against the sum of mRSS values (total mRSS) from the same body sites for that subject. The line represents a linear fit.

Figure 2(a) is a heatmap of values of the area under the ROC curve of a binary classifier characterizing healthy controls versus SSc patients across wavelength and spatial frequency. The highest AUC values are seen at 811 nm and $0.2\;{\mathrm{mm}}^{-1}$, 851 nm and $0.15\;{\mathrm{mm}}^{-1}$, and 851 nm and $0.2\;{\mathrm{mm}}^{-1}$, with AUC values 0.88 to 0.89. These AUC values are not significantly different from each other (DeLong’s test, p>0.01), indicating that they have comparable performance. Figure 2(b) shows example measurements at 851 nm and $0.15\;{\mathrm{mm}}^{-1}$. Each point in the boxplot represents the $R_{d}$ value at a given measurement site. There is a statistically significant difference between the patient and control groups at this $\lambda \text{-}f_{x}$ combination (p>0.0001, Wilcoxon rank sum test), which is also observed at the other $\lambda \text{-}f_{x}$ combinations with high AUC values (e.g., 811 nm and $0.2\;{\mathrm{mm}}^{-1}$, 811 nm and $0.15\;{\mathrm{mm}}^{-1}$, and 851 and $0.2\;{\mathrm{mm}}^{-1}$).

Figure 2(c) is a heatmap of Spearman’s correlation coefficients between the sum of $R_{d}$ measurements and the sum of the corresponding mRSS values. Here, a peak is seen at a $\lambda \text{-}f_{x}$ combination of 811 nm and $0.2\;{\mathrm{mm}}^{-1}$ and 851 nm and $0.15\;{\mathrm{mm}}^{-1}$, corresponding to a correlation coefficient of 0.60. A correlation coefficient of 0.57 is seen for $\lambda \text{-}f_{x}$ combinations of 851 nm and $0.1\;{\mathrm{mm}}^{-1}$, 851 nm and $0.2\;{\mathrm{mm}}^{-1}$, 811 nm and $0.1\;{\mathrm{mm}}^{-1}$, and 811 nm and $0.15\;{\mathrm{mm}}^{-1}$. An example of this correlation is visualized in Fig. 2(d) at 851 nm and $0.15\;{\mathrm{mm}}^{-1}$. Each point represents the total mRSS plotted against the total $R_{d}$ value for a given SSc patient.

The peaks in AUC and correlation coefficient at the wavelengths (λ) of 811 and 851 nm and spatial frequencies ($f_{x}$) of 0.15 and $0.2\;{\mathrm{mm}}^{-1}$ indicate that these parameters are optimal for detecting changes occurring in the skin during the progression of SSc. It is interesting to note here that these two metrics represent different ways of assessing the disease. The AUC measures how the parameters differentiate the skin of healthy controls from SSc patients, whereas the correlation with mRSS measures the extent of disease progression within the patient group.

**Fig. 3:**
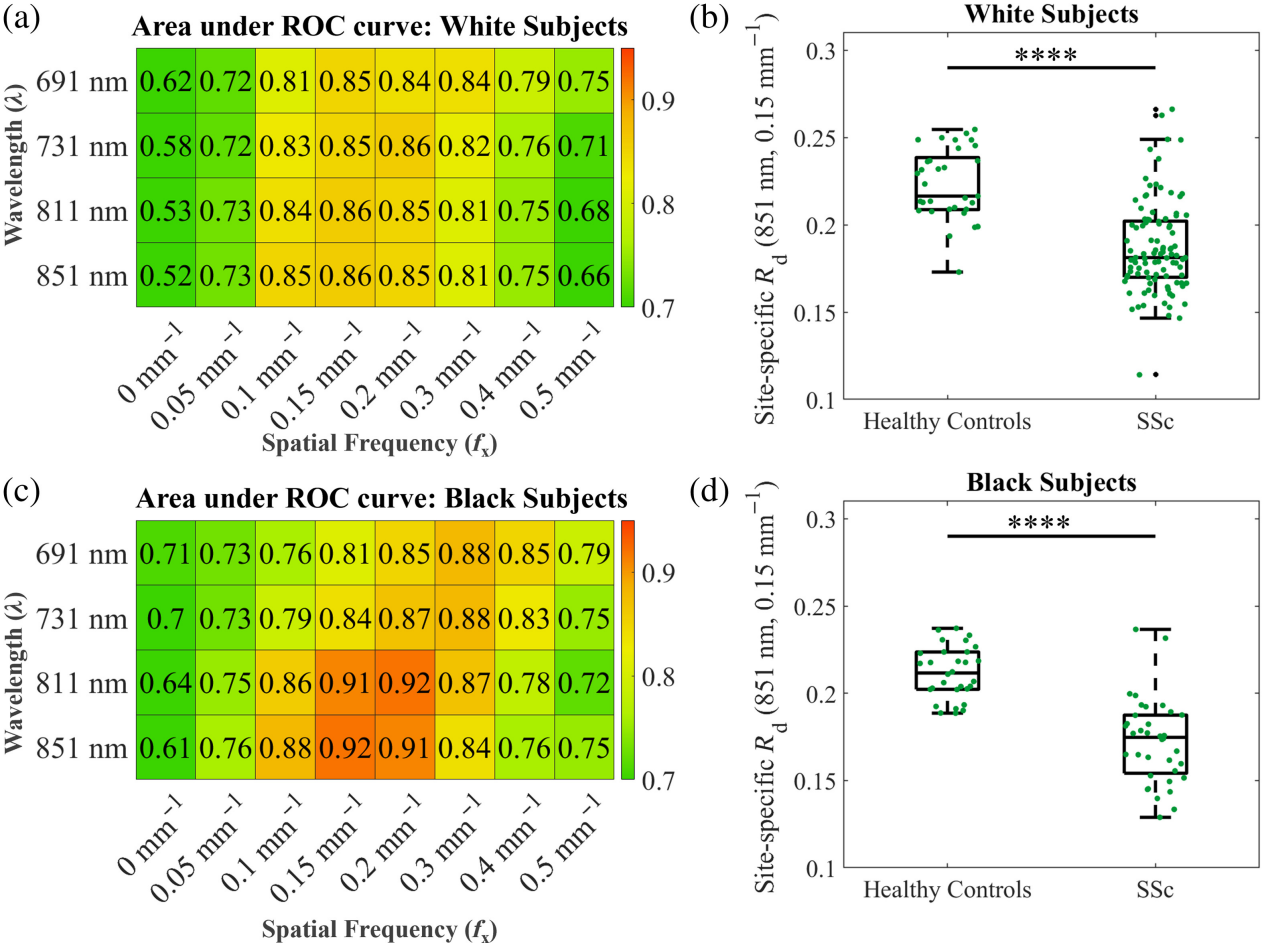
(a) Heatmap of AUC values for site-specific $R_{d}$ values from healthy controls and SSc patients for subjects who self-identify as White, irrespective of ethnicity. (b) Site-specific $R_{d}$ values of self-identifying White healthy controls and SSc patients for 851 nm and $0.15\;{\mathrm{mm}}^{-1}$. Each point on the boxplot represents the $R_{d}$ value of a different measurement site. (****p<0.0001, Wilcoxon rank sum test). (c) Heatmap of AUC values for subjects who self-identify as Black, irrespective of ethnicity. (d) Site-specific $R_{d}$ values of self-identifying Black healthy controls and SSc patients at 851 nm and $0.15\;{\mathrm{mm}}^{-1}$ (****p<0.0001, Wilcoxon rank sum test).

Clinical data were stratified by race into patients self-reporting as White or Black, irrespective of ethnicity. Figure 3(a) shows the heatmap for the AUC values for all subjects identifying as White. High AUC values were observed across all wavelengths (691 to 851 nm), peaking at the $f_{x}$ values of 0.15 and $0.2\;{\mathrm{mm}}^{-1}$. These $f_{x}$ values perform well at separating patients from controls irrespective of wavelength, with AUC values ranging from 0.85 to 0.86. There was also a statistically significant difference between the site-specific $R_{d}$ values at 851 nm and $0.15\;{\mathrm{mm}}^{-1}$ [Fig. 3(b), Wilcoxon rank sum test, ****p<0.0001].

Figure 3(c) shows the heatmap for AUC values for subjects identifying as Black. Unlike the White subjects, the optimal $f_{x}$ value decreased at the longer measured wavelengths. The best AUC values were observed at 811 nm and $0.2\;{\mathrm{mm}}^{-1}$ and 851 nm and $0.15\;{\mathrm{mm}}^{-1}$. These AUC values were not statistically significantly different (DeLong’s test, p>0.01), indicating these $\lambda \text{-}f_{x}$ combinations have comparable performance. There was a statistically significant difference between site-specific $R_{d}$ values for Black patients and controls at 851 nm and $0.15\;{\mathrm{mm}}^{-1}$ [Fig. 3(d)], Wilcoxon rank sum test, ****p<0.0001). This subanalysis suggests that subjects who self-identify as Black are somewhat more separable into SSc patients and healthy controls compared with White subjects. However, the best separation of SSc patients from healthy controls across both Black and White subgroups occurred at the previously identified “optimal” $\lambda \text{-}f_{x}$ pairs of 811 nm and $0.2\;{\mathrm{mm}}^{-1}$, 851 nm and $0.15\;{\mathrm{mm}}^{-1}$, and 851 nm and $0.2\;{\mathrm{mm}}^{-1}$. Example ROC curves for both groups of subjects at example optimal imaging parameters and nonoptimal imaging parameters are seen in Fig. S2 in the Supplementary Material.

### 3.3 Monte Carlo Results

**Fig. 4:**
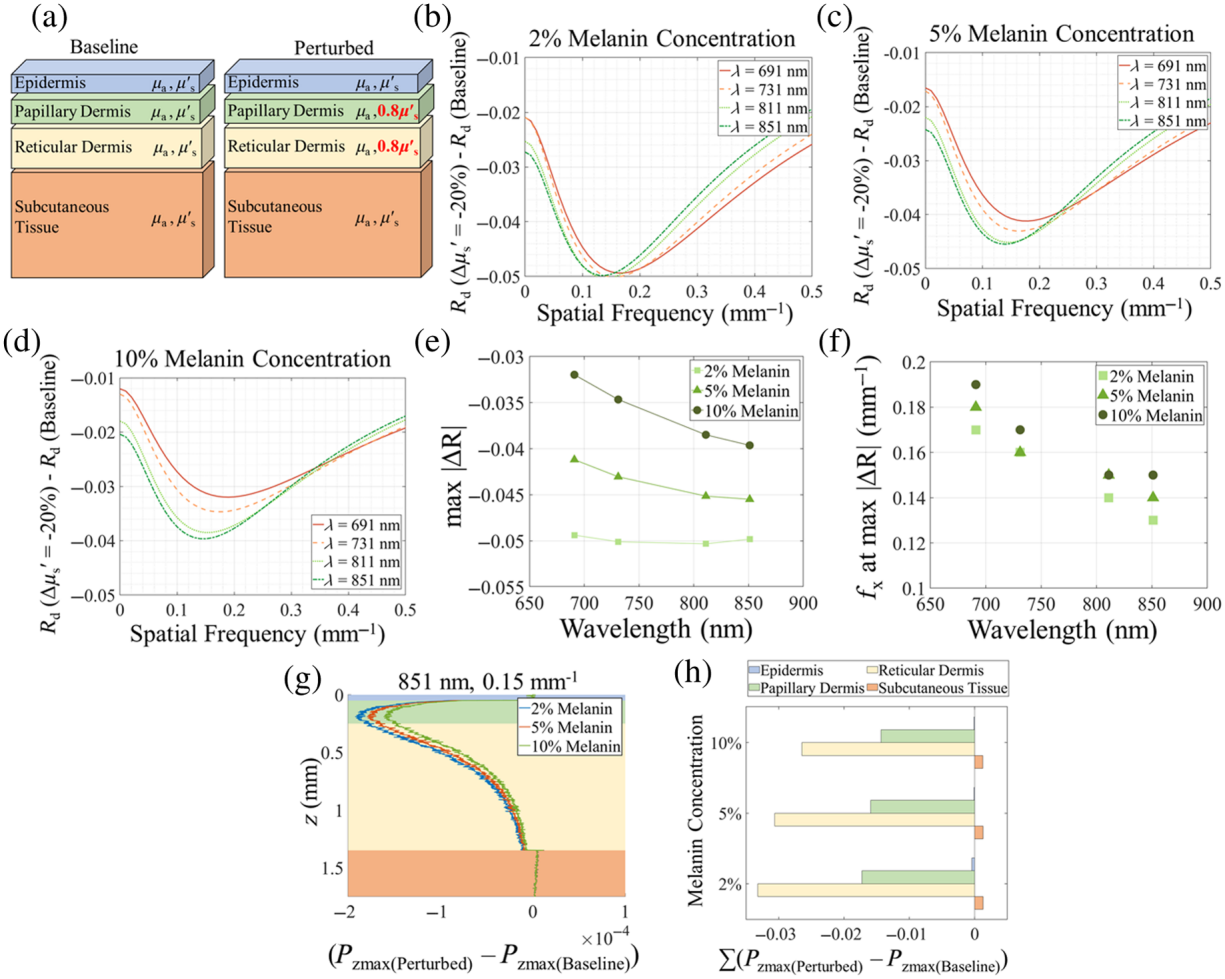
(a) The multilayer skin model used for Monte Carlo simulations. “Perturbed” optical properties refer to a reduction of the scattering coefficient of the papillary dermis and the reticular dermis by 20%, as shown in the bold red text. (b)–(d) Change in simulated measured reflectance between the perturbed model and the baseline model as a function of spatial frequency for four wavelengths. Simulations were performed for different melanin concentrations: 2% (b), 5% (c), and 10% (d). (e) Values of the maximum absolute change in measured $R_{d}$ across all simulated spatial frequencies between perturbed model and baseline models as a function of wavelength. (f) Spatial frequencies at which the absolute change in the measured $R_{d}$ between the perturbed model and baseline models is maximized (indicating higher sensitivity to scattering changes in the dermis), plotted as a function of wavelength for a range of melanin concentrations. (g) Difference between the $P_{z_{\max}}$ values from the perturbed and baseline models plotted as a function of depth (z). Layers of the skin are represented by the shading (blue: epidermis, green: papillary dermis, yellow: reticular dermis, orange: subcutaneous tissue). (h) The difference in the integral of the $P_{z_{\max}}$ values from the perturbed model and baseline models, taken over the z-values corresponding to the four layers of the skin, plotted for each simulated melanin concentration (2%, 5%, and 10%). These values represent the contribution of each layer to the measured change in reflectance shown at 851 nm and $0.15\;{\mathrm{mm}}^{-1}$.

Figure 4(a) provides a schematic of the four-layer skin model that was used for Monte Carlo simulations. The baseline model was assigned optical properties that represented healthy skin, as in our previous work,^1^ whereas the perturbed model represented sclerodermatous skin. The $\mu_{s}^{\prime }$ values used for the papillary and reticular dermis were decreased by 20% in the perturbed model. Simulated reflectance ($R_{d}$) values for all simulated wavelengths and frequencies from the perturbed model were compared with $R_{d}$ values from the baseline model. The $\lambda \text{-}f_{x}$ pairs that corresponded to the maximum differences between $R_{d}$ values from the baseline and perturbed models were deemed the most sensitive imaging parameters to changes in dermal scattering. These are compared with the optimum $\lambda \text{-}f_{x}$ pairs from our clinical data analysis.

Trends seen in the results of the Monte Carlo simulations align well with the trends seen in the clinical data. Figure 4(b) shows the $R_{d}$ change as a function of $f_{x}$ for a 2% epidermal melanin concentration. Like in the clinical data for White subjects [Fig. 3(a)], we see the largest $R_{d}$ change (most negative values) falls between $f_{x}$ values of 0.1 to $0.2\;{\mathrm{mm}}^{-1}$ across all wavelengths. The wavelength dependence of the $R_{d}$ change becomes more pronounced in the 5% and 10% melanin concentration cases [Figs. 4(c) and 4(d), respectively]. In both these cases, we see that 811 and 851 nm provide a larger $R_{d}$ change as compared with 691 and 731 nm. Similar trends were observed in the clinical data for Black patients, in which the wavelength dependence was pronounced [Fig. 3(c)].

Figure 4(e) shows the $R_{d}$ change as a function of wavelength for different melanin concentrations. Here, the magnitude of $R_{d}$ change varies considerably between low and high melanin concentrations at shorter wavelengths but exhibits less variation with melanin concentration at longer wavelengths. Figure 4(f) shows the $f_{x}$ values that correspond to the maximum absolute change in $R_{d}$. As expected, these optimal $f_{x}$ values are between 0.1 and $0.2\;{\mathrm{mm}}^{-1}$, with a decrease in optimal $f_{x}$ at longer wavelengths. This decrease in optimal $f_{x}$ at longer wavelengths may seem surprising at first. Given that shorter wavelengths generally have shallower penetration depths in tissue compared with longer wavelengths, we might expect the optimal $f_{x}$ to be lower at shorter wavelengths. However, this trend can be explained by considering the spectral variations in the transport mean free path. The significant increase in the dermal transport mean free path from 691 nm (mean free path = 0.35 mm) to 851 nm (mean free path = 0.46 mm) leads to a lower spatial frequency at which the largest sensitivity to scattering perturbation occurs. This is discussed in part 1 of this two-part publication (see Fig. 5 of Ref. 1).

Figures 4(g) and 4(h) provide insight into the tissue probing depth and show depth statistics for a simulation at 851 nm and $0.15\;{\mathrm{mm}}^{-1}$. Our Monte Carlo simulations provide values of $P_{z_{\max}}$ (z), or the “$z_{\text{max}}$” distribution, which quantifies the detected photon packets that reach no further than a maximal depth “z” in the multilayer model.^24^ $P_{z_{\max}}(z)$ can be integrated across all depths to provide the total simulated diffuse reflectance $R_{d}$. Figure 4(g) plots the difference between $P_{z_{\max}}$ (z) in the perturbed case and the $P_{z_{\max}}(z)$ in the baseline case for all z values. The figure shows the depths that contribute to the largest change in $R_{d}$.

From Fig. 4(g), it is apparent that the largest changes occur in the papillary dermis and the top half of the reticular dermis. The changes follow the same trend for different melanin concentrations. However, for the 2% melanin case, there are larger perturbations as there are fewer photons lost to melanin absorption compared with the 10% case.

Figure 4(h) shows the difference in $P_{z_{\max}}(z)$ between the baseline and the perturbed state, summed over all the z values that represent each skin layer. Here, the epidermis and the subcutaneous tissues provide minimal contributions to the difference between the perturbed and the baseline models, whereas the papillary and the reticular dermis have a large contribution. Once again, increased melanin concentrations (5%, 10%) result in a lower overall $R_{d}$, and hence a lower overall $P_{z_{\max}}$ value, compared with a lower melanin concentration (2%). Overall, the papillary and reticular dermis layers are the principal sources of contrast between the perturbed and the baseline models.

## 4 Discussion

In this work, we have demonstrated the potential of SLIM as a method to quantify skin involvement in SSc. By analyzing clinical measurements and comparing these results to Monte Carlo simulations, we identified optimal $\lambda \text{-}f_{x}$ pairs that provide the largest reflectance changes in response to structural alterations occurring in the papillary and reticular dermis and maximally discriminate between healthy controls and SSc patients. This potentially provides the capability to objectively diagnose and quantify the extent of skin involvement in SSc. Given that the current gold standard, manual palpation (mRSS), has multiple limitations, including subjectivity and high interobserver variability, SLIM may represent a reliable, objective, and quantitative alternative. In addition, as SLIM requires fewer measurements, minimal postprocessing, and no inverse model, it may provide a simpler alternative to SFDI as an objective means to assess SSc.

In our clinical data, $R_{d}$ measurements in the λ range of 811 to 851 nm and $f_{x}$ range of 0.15 to $0.2\;{\mathrm{mm}}^{-1}$ were found to be most sensitive to changes in sclerodermatous skin. Moreover, when clinical data were stratified by self-reported race, longer wavelengths performed better (i.e., provided higher AUC) for subjects who self-reported as Black, whereas all wavelengths performed similarly for patients who self-reported as White. This effect is broadly attributed to the fact that melanin absorption decreases with wavelength.^25^^–^^27^ These clinical observations were largely recapitulated in the simulation results, where the $\Delta R_{d}$ values were largest at long wavelengths at high melanin concentrations. Depth statistics from simulations indicated that measurements at key optimal $\lambda \text{-}f_{x}$ pairs, such as 851 nm and $0.15\;{\mathrm{mm}}^{-1}$, were highly sensitive to changes in both the papillary and reticular dermis. Importantly, if one desires to identify a single $\lambda \text{-}f_{x}$ pair that optimizes reflectance changes across skin tone, both our clinical measurements and simulation results suggest $\lambda \text{-}f_{x}$ pairs in the following ranges: λ≈811 to 851 nm and $f_{x}\approx 0.15$ to $0.2\;{\mathrm{mm}}^{-1}$.

**Fig. 5:**
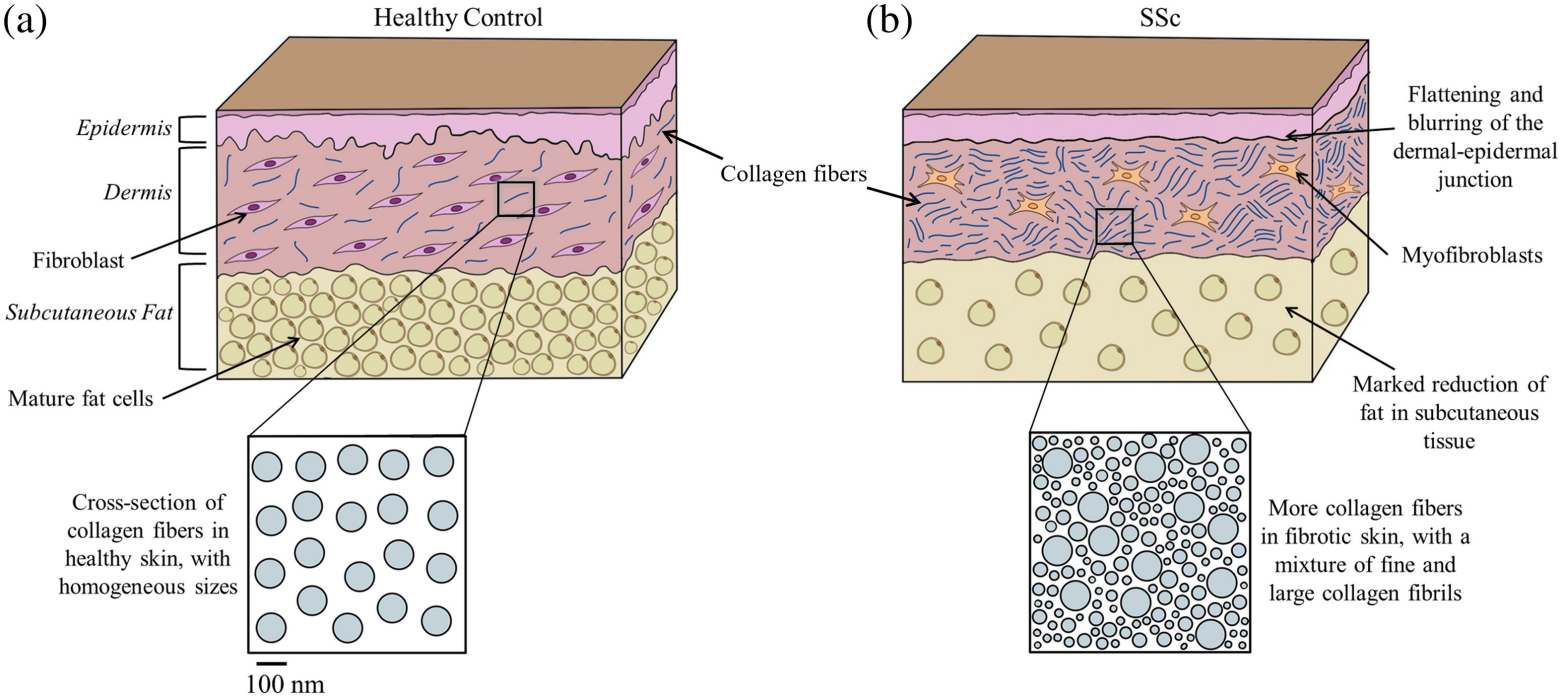
Summary of biological changes in sclerodermatous skin (b), compared with healthy skin (a).

The hypothesis that the changes in $R_{d}$ measured by SLIM are due to biological changes occurring in both the papillary and reticular dermis is supported by the concordance between our clinical and simulation results, where we assumed a scattering reduction throughout the dermis. We further support this hypothesis by reviewing the known biological changes that occur in sclerodermatous skin, which are summarized in Fig. 5 and below.

First, there is a flattening of the dermal-epidermal junction (DEJ) and a loss of structure of the rete ridges.^3^^,^^28^ This is also seen in prior work with optical coherence tomography (OCT) in SSc, where there is a “loss of visualization” of the DEJ, which looks blurred in the OCT images.^29^ Two-photon microscopy images of SSc biopsies also report a flattening of the rete ridges in the DEJ.^30^ Second, there is a marked increase in dermal collagen content in sclerodermatous skin.^31^ Fibroblasts derived from sclerodermatous skin were shown to synthesize more collagen than normal controls, and exaggerated collagen production is associated with fibroblasts derived from the lower dermis.^32^ The dermis is known to thicken by broad, elongated bundles of homogenized collagen oriented parallel to surface epithelium.^19^ The dermal collagen is tightly packed,^16^ and collagen fibrils of a smaller diameter are observed throughout the dermis and near the basal lamina of the DEJ.^16^ Young collagen fibers are also seen in the dermis and subcutaneous tissue.^16^ Collagen fibrils from patients with SSc have been shown to have a mean diameter of 40 to 50 nm, which is considerably smaller than the mean fibril diameter of 120 nm found in normal adults.^16^ These thinner fibrils have the same cross-banding patterns and fine structure as the fibrils found in human fetal collagen fibrils, or embryonic collagen, which also has a mean diameter of 40 to 50 nm.^20^ Finally, there is a marked reduction of fat and atrophy of the subcutaneous tissue, which is replaced by newly synthesized connective tissue.^33^ As the disease progresses, the subcutaneous tissue becomes thinner and the dermis thickens.^31^

Taken together, we surmise that the biological changes driving the decrease in $R_{d}$ observed at the optimal $\lambda \text{-}f_{x}$ pairs identified in this work are likely both changes at the DEJ and the decrease in fiber and fibril diameter in the newly deposited collagen in the dermis. Embryonic collagen fibers and fibrils have been shown to have a lower scattering coefficient than mature collagen, with the scattering coefficient of fibrils increasing linearly with gestational maturity.^21^ This matches the clinical observations in our previous work where we reported a reduction in the reduced scattering coefficient in SSc patients compared with healthy controls.^2^ It also matches the clinical data presented in this paper, where there is a decrease in $R_{d}$ of SSc patients compared with healthy controls.

In our previous work, we investigated the potential of SFDI as an alternative to mRSS.^2^ Here, we have introduced SLIM as a simpler alternative to SFDI for assessing skin in SSc. SLIM is similar to SFDI but differs in some important ways and has some key advantages over SFDI. Most frequently, SFDI uses $R_{d}$ values from two different spatial frequencies, typically one $R_{d}$ value from a lower spatial frequency (most often, DC or $0\;{\mathrm{mm}}^{-1}$) and one from a higher spatial frequency to extract optical properties ($\mu_{a}$ and $\mu_{s}^{\prime }$) per pixel using an inversion algorithm. This inversion algorithm is often time-consuming and computationally intensive, which provides challenges to real-time measurements, especially when multiple layers are considered.^34^ Moreover, it is associated with uncertainty in the extraction of optical properties.^35^ When extracting optical properties, information is convolved from the two spatial frequencies that sample different tissue depths and confound the spatial assignment of the recovered properties. Accordingly, one must consider the differing depth sensitivities within a sample between different spatial frequencies, which can lead to a partial volume effect where the shallower penetrating spatial frequency is only partially sensitive to the sample volume interrogated by the deeper penetrating spatial frequency. Consequently, this partial volume mismatch could obfuscate important tissue information, especially in diseases where key biological changes manifest at specific depths in a tissue volume as is the case with SSc. SLIM, however, measures reflectance at a single spatial frequency and therefore requires considerably less postprocessing compared with SFDI and does not convolve information from disparate tissue depths in the same manner as SFDI.

To more fully compare SFDI and SLIM, the performance of $\mu_{s}^{\prime }$ for assessing sclerodermatous skin is shown for all subjects, as well as race-stratified data, in Fig. S2 in the Supplementary Material. We see similar correlational strength for $\mu_{s}^{\prime }$ (SFDI, Fig. S3B in the Supplementary Material) and $R_{d}$ [SLIM, Fig. 2(c)] when compared with mRSS scores. In addition, there is no statistical difference in the ROC curves for most of the best-performing optical parameters for SLIM versus SFDI (DeLong’s test, p>0.01). This shows that although SFDI and SLIM perform with almost equivalent performance, SLIM enjoys the advantage of requiring fewer measurements and lower computational overhead as no inverse model is needed.

This work has several limitations. First, with only 25 SSc patients and 18 controls, the clinical data set is limited. Although the patients measured range across the progression of the disease, there is not a large enough sample size to stratify the patients based on their individual site mRSS scores. Based on disease progression, there could be different optimal imaging parameters that could assess the skin involvement, but we are unable to assess this with our current data set. Second, although we use self-reported race to stratify our subject population into White and Black, this is far from an objective measure of melanin concentration. Future work will include individual topology angle measurements with a colorimeter, which will provide a more objective metric of skin tone. Third, although SSc progresses across the entire body, and mRSS is measured at 17 different body locations, SLIM measurements are only collected on six body locations, the sites where positioning under the commercial system was easy and comfortable for the subjects. Fourth, in this work, we compare our results with mRSS, which is an admittedly lacking gold standard.^13^^–^^15^ Future work will calculate correlations with histopathology metrics, which will provide a more reliable metric for SSc progression and also help identify at what depths the changes under the skin are happening through microscopy of skin biopsies. Finally, the multilayer skin model used in the Monte Carlo simulations in this work is a simplified representation of biological changes occurring within the skin during SSc disease progression. It does not consider changes to the thickness of skin layers during disease progression, which have been mentioned in prior literature.^31^ Although the results from the simulations have similar trends to the clinical data, the model choices of optical properties, layer thicknesses, magnitude, depth, and volume of the applied scattering perturbation have a marked impact on the results of the simulation and, hence, on the optimal $\lambda \text{-}f_{x}$ combination that is most sensitive to said perturbation. This is exemplified in part 1 of this work,^1^ where perturbations confined just to the papillary dermis led to different optimal imaging parameters, with simulated photons being sensitive to different depths within the skin.

## 5 Conclusion

In summary, we demonstrate the clinical application of structured light imaging mesoscopy (SLIM) as a method to evaluate SSc skin involvement. By using an optimal spatial frequency/wavelength combination, the depths of relevant biological changes can be targeted. SLIM instrumentation and processing are considerably simplified compared with that of SFDI, and this technique can potentially be clinically translated to help clinicians assess skin involvement, improve patient outcomes, and aid with research and clinical trials for treatments for SSc.

## Supplementary Material

10.1117/1.BIOS.2.2.025002.s01

## Data Availability

Data points contained in the figures of this paper are included in the Supplementary Material.
