# Supplementary material for: Structured light imaging mesoscopy: application to skin changes in scleroderma
Source: Biophotonics Discov. 2025 Jun 18;2(2):025002. doi: 10.1117/1.BIOS.2.2.025002 (PMC13098738; doi:10.1117/1.BIOS.2.2.025002)
Supplement: Supplementary file 1 [file BIOS_002_025002_SD001.pdf]

Structured Light Imaging Mesoscopy Part 2: Application to Skin Changes in Scleroderma

Aarohi Mahesh Mehendale[a], Mahsa Parsanasab[b,c], Kavon Karrobi[a], Hung Vo[d,e], Andreea M. Bujor[d,e], Vasan Venugopalan[b,c], Darren Roblyer[a,f*]

a) Boston University, Department of Biomedical Engineering, Boston, MA

b) University of California, Irvine, Department of Chemical and Biomolecular Engineering, Irvine, CA

c) University of California, Irvine, Beckman Laser Institute and Medical Center, Irvine, CA

d) Boston University Chobanian & Avedisian School of Medicine, Division of Rheumatology, Boston, MA

e) Boston University, Arthritis and Autoimmune Diseases Center, Boston, MA

f) Boston University, Department of Electrical and Computer Engineering, Boston, MA

*E-mail: roblyer@bu.edu

Supplementary Figures and Tables

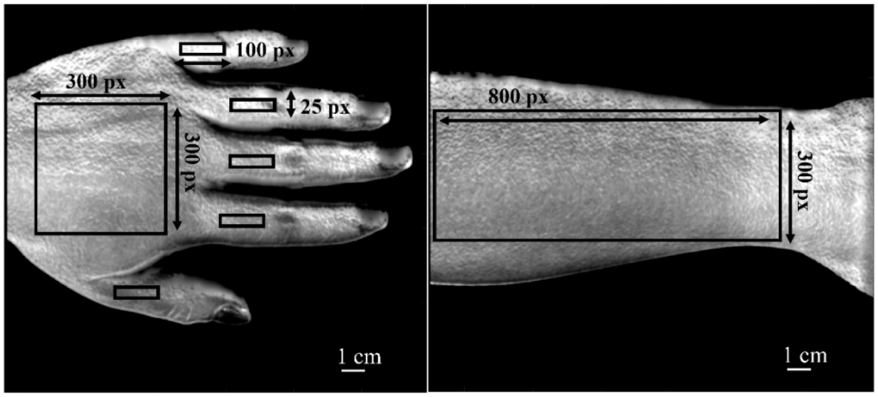

Figure S1: *Anatomic locations and sizes of selected regions of interest on an example subject's left hand and forearm.*

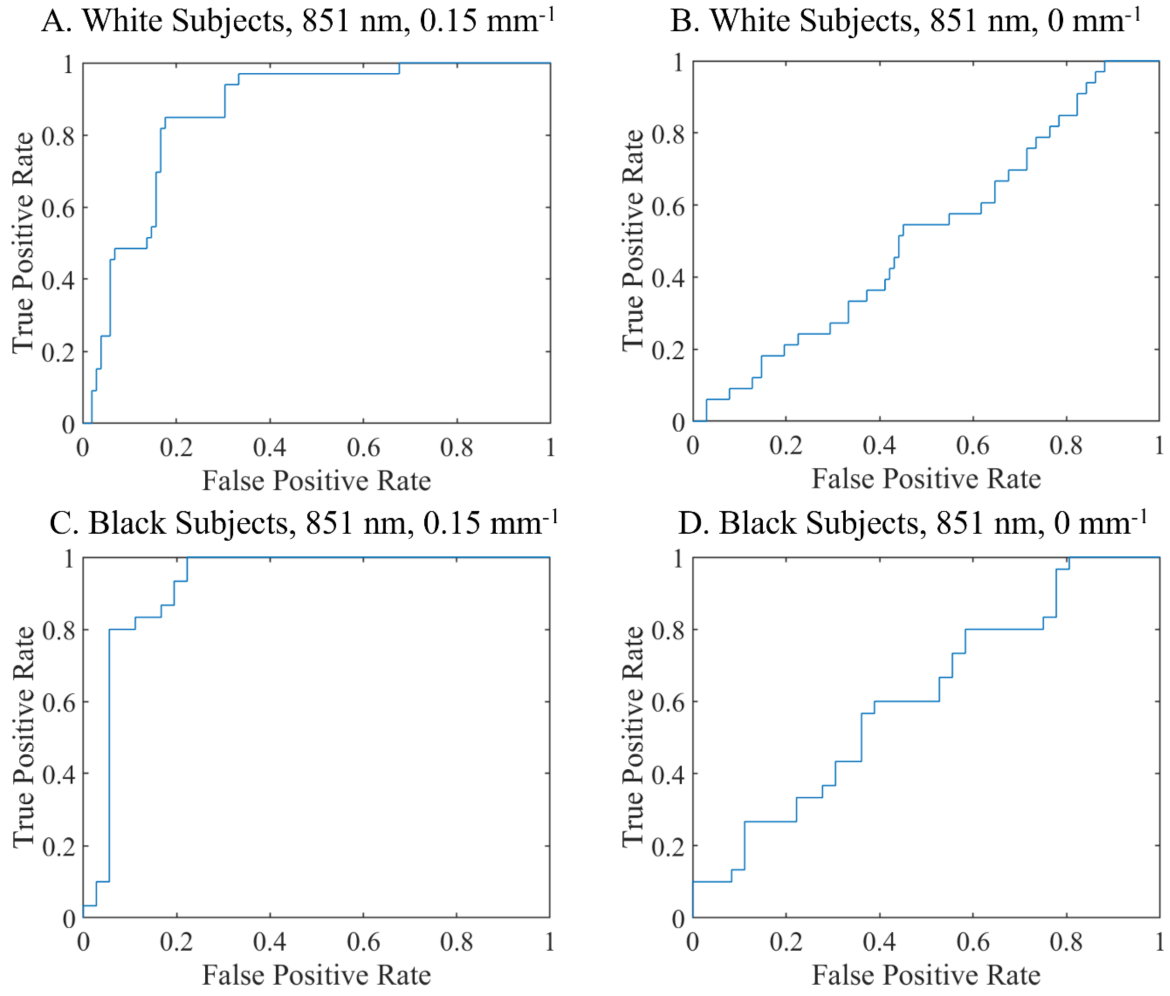

Figure S2*: Example ROC curves for white (A, B) and black (C, D) subjects, at the optimal imaging parameters (851 nm and 0.15 mm^{-1}) (A, C) and another example imaging parameter (851 nm and DC or 0 mm^{-1}) (B, D).*

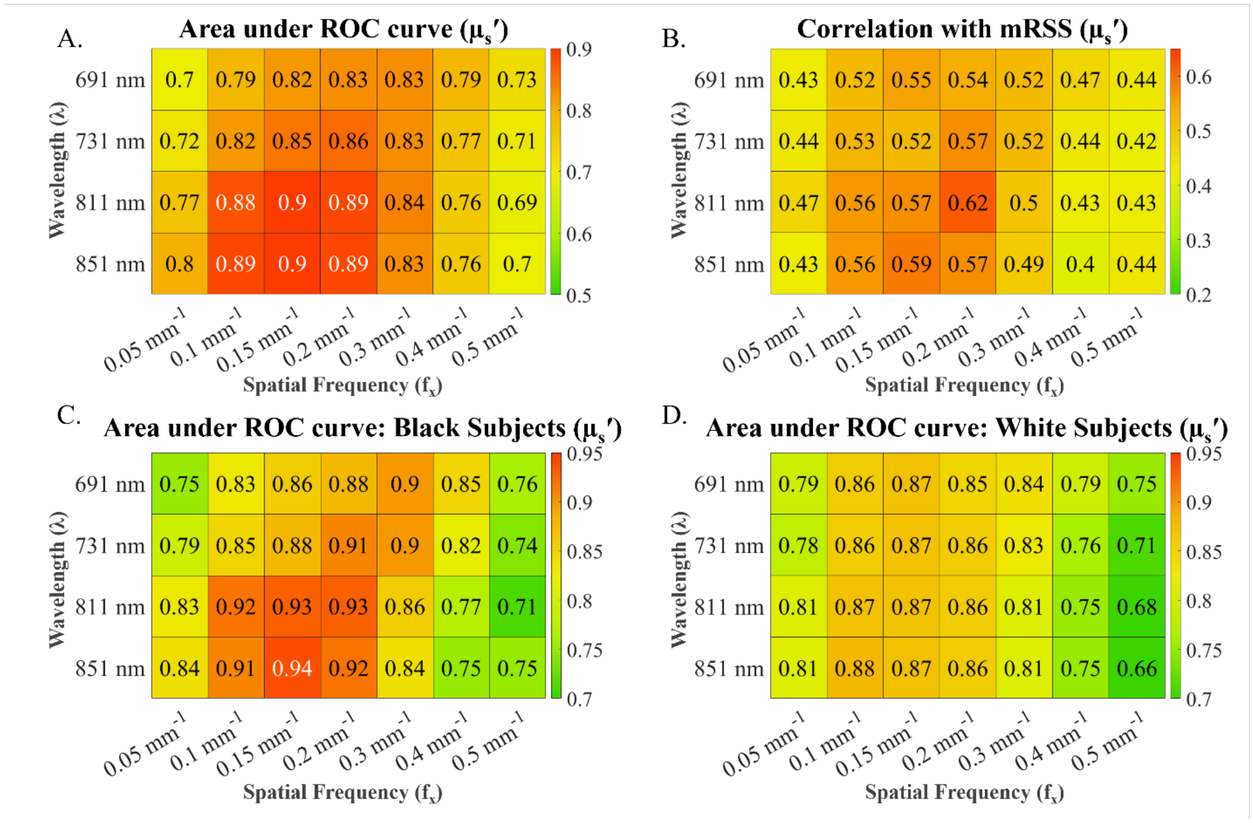

Figure S2A. *Heatmap of area under ROC curves of threshold classification between site-specific μ_s' values for all healthy controls and SSc patients, at four wavelengths and calculated with a combination of DC and seven non-zero spatial frequencies. Area under the ROC curve is used as a surrogate for separability of the two groups. Red represents a higher area under the curve (high separability), and green represents a lower area under the curve (lower separability)*

Figure S2B. *Heatmap of Spearman's correlation coefficients between total mRSS and total μ_s' across six measured sites. Correlations are calculated between total mRSS and total μ_s' at four wavelengths and calculated at a combination of DC and seven non-zero spatial frequencies. Red represents a higher correlation, and green represents a lower correlation.*

Figure S2C. *Heatmap of area under ROC curves of threshold classification between site-specific μ_s' values for healthy controls and SSc patients that self-identify as black, at four wavelengths and calculated with a combination of DC and seven non-zero spatial frequencies. Area under the ROC curve is used as a surrogate for separability of the two groups. Red represents a higher area under the curve (high separability), and green represents a lower area under the curve (lower separability)*

Figure S2D. *Heatmap of area under ROC curves of threshold classification between site-specific μ_s' values for healthy controls and SSc patients that self-identify as white, at four wavelengths and calculated with a combination of DC and seven non-zero spatial frequencies. Area under the ROC curve is used as a surrogate for separability of the two groups. Red*

represents a higher area under the curve (high separability), and green represents a lower area under the curve (lower separability)

Table S1: Baseline optical properties of the multi-layer skin model across all simulated wavelengths, for a melanin concentration of 0%

	**μ_a (691 nm)**	**μ_a (731 nm)**	**μ_a (811 nm)**	**μ_a (851 nm)**
Epidermis	0.0001	0.0003	0.0004	0.0008
Papillary Dermis	0.0071	0.0071	0.0104	0.0136
Reticular Dermis	0.0071	0.0071	0.0104	0.0136
Subcutaneous Tissue	0.0239	0.0215	0.0329	0.0402
	**μ'_s (691 nm)**	**μ'_s (731 nm)**	**μ'_s (811 nm)**	**μ'_s (851 nm)**
Epidermis	4.4621	4.1660	3.7032	3.5200
Papillary Dermis	2.8515	2.6374	2.3059	2.1760
Reticular Dermis	2.8515	2.6374	2.3059	2.1760
Subcutaneous Tissue	1.1548	1.1008	1.0125	0.9755

Table S2: Optical properties of the perturbed multi-layer skin model across all simulated wavelengths, for a melanin concentration of 0%

	**μ_a (691 nm)**	**μ_a (731 nm)**	**μ_a (811 nm)**	**μ_a (851 nm)**
Epidermis	0.0001	0.0003	0.0004	0.0008
Papillary Dermis	0.0071	0.0071	0.0104	0.0136
Reticular Dermis	0.0071	0.0071	0.0104	0.0136
Subcutaneous Tissue	0.0239	0.0215	0.0329	0.0402
	**μ'_s (691 nm)**	**μ'_s (731 nm)**	**μ'_s (811 nm)**	**μ'_s (851 nm)**
Epidermis	4.4621	4.1660	3.7032	3.5200
Papillary Dermis	2.2812	2.1099	1.8447	1.7408
Reticular Dermis	2.2812	2.1099	1.8447	1.7408
Subcutaneous Tissue	1.1548	1.1008	1.0125	0.9755

Table S3: Values of epidermis absorption coefficient for various simulated melanin concentrations, across simulated wavelengths

Epidermis μ_a	**μ_a (691 nm)**	**μ_a (731 nm)**	**μ_a (811 nm)**	**μ_a (851 nm)**
Melanin = 2%	0.4469	0.3677	0.2563	0.2172
Melanin = 5%	1.1171	0.9187	0.6402	0.5419
Melanin = 10%	2.2340	1.8370	1.2799	1.0830
